# Exploring drivers and management strategies of informal patient payments in hospitals: A qualitative study in Iran

**DOI:** 10.1371/journal.pone.0349768

**Published:** 2026-05-22

**Authors:** Ali Reza Yusefi, Jamshid Bahmaei

**Affiliations:** 1 National Center for Health Insurance Research, Tehran, Iran; 2 Student Research Committee, Sirjan School of Medical Sciences, Sirjan, Iran; 3 Department of Public Health, Behbahan Faculty of Medical Sciences, Behbahan, Iran; Simon Diedong Dombo University of Business and Integrated Development Studies, GHANA

## Abstract

**Background:**

Informal patient payments remain a persistent and multifaceted challenge in many health systems, particularly in low- and middle-income countries. These payments, often made outside official channels, can undermine equity, transparency, and trust in healthcare delivery. This qualitative study aimed to explore the underlying causes of informal payments and identify feasible management strategies from the perspectives of key stakeholders.

**Methods:**

This applied study was conducted using a qualitative method and semi-structured interviews between September 10, 2024, and January 17, 2025. A total of 31 participants, including senior policymakers from the Ministry of Health, hospital managers, medical specialists, nursing managers, hospital supervisors, health economics and healthcare management faculty members, and patients with experience of informal payments in Iranian hospitals, were selected through purposive and snowball sampling methods. Latent content analysis was employed for data analysis, and MAXQDA software (version 2022) was used to extract the main and subcategories.

**Results:**

Six main themes and 39 subcategories were identified regarding the causes of informal patient payments. From the perspectives of policymakers, physicians, and service providers, four main themes with 27 subcategories emerged, including factors related to salaries and benefits, structural and organizational issues, laws and regulations, and ethical and cultural considerations. From the perspective of service recipients, two main themes with 12 subcategories were identified, covering service-related and cultural factors. Regarding management strategies to address informal payments, three main themes with 14 subcategories were found, encompassing structural, legal, and cultural approaches.

**Conclusion:**

Informal payments in hospitals are driven by a complex interplay of economic, structural, legal, cultural, and ethical factors. Addressing this issue requires a multifaceted strategy, including tariff reform, strengthening legal and regulatory frameworks, promotion of professional ethics, improved service delivery systems, and public awareness campaigns. Sustainable reduction of informal payments demands coordinated efforts across all levels of the health system.

## Background

Informal payments in the healthcare sector have become a major concern for health systems, particularly in low- and middle-income countries (LMICs), where institutional weaknesses, limited financial resources, and inadequate monitoring mechanisms prevail [1,2]. These payments, often made directly by patients to healthcare providers outside official channels, can take various forms, cash, gifts, or services, and are typically exchanged to ensure access, accelerate treatment, or receive better quality care [3,4]. Informal payments are distinct from official co-payments or user fees; they are neither regulated by the healthcare system nor recorded in financial accounts, making them difficult to track and manage [5,6]. Although these transactions are often perceived as cultural norms or “tokens of gratitude,” in practice they constitute a major threat to the principles of equity, transparency, and efficiency in healthcare delivery [7].

The issue of informal payments is particularly critical in hospital settings, where patients and families may experience greater vulnerability, urgency, and dependency on medical staff [8]. Hospitals, as complex institutions with high volumes of patient-provider interactions and technical procedures, are especially susceptible to unofficial financial exchanges [9]. When patients are required or encouraged to make informal payments, it creates inequitable access to services, disproportionately disadvantaging those with fewer financial means [10].

Furthermore, such payments can contribute to corruption, lower provider morale, and erode public trust in the healthcare system [11]. In some cases, they may even lead to a dual-track system, where those who pay unofficially receive faster or better care, while others endure delays or neglect [12].

The persistence of informal payments in the Iranian healthcare system reflects broader structural and policy challenges [13]. Although Iran has undertaken health sector reforms in recent years, including the Health Transformation Plan (HTP), which aimed to reduce out-of-pocket expenditures and improve service quality, informal payments continue to be reported across various levels of care, particularly in specialized and inpatient settings [14,15]. A range of factors may be contributing to this ongoing phenomenon: insufficient public health funding, unclear boundaries between public and private services, inadequate provider compensation, and weak enforcement of regulations [16,17]. Cultural and social norms may also play a role, as patients and families often feel obligated to provide additional payments to express gratitude or secure better care [18]. However, existing policies have failed to address the deeper institutional and behavioral roots of the issue.

While several quantitative studies have documented the prevalence and economic burden of informal payments in Iran and other LMICs, there is a notable lack of qualitative research that explores the lived experiences, perceptions, and motivations of stakeholders involved. Understanding the nuanced drivers of informal payments, why patients feel compelled to pay, why providers accept them, and how hospital managers navigate such practices, requires an in-depth, contextual approach that goes beyond numerical data.

Moreover, there is limited evidence on the specific management strategies that could be effective in mitigating informal payments in different institutional contexts. This study seeks to fill this critical knowledge gap by conducting a qualitative investigation into the causes of informal payments among patients in Iranian hospitals and by identifying practical strategies for managing and potentially reducing such practices. Therefore, the objective of this study was to explore the drivers of informal patient payments in Iranian hospitals and to identify feasible management strategies from the perspectives of key stakeholders. By focusing on stakeholders’ lived experiences and perspectives, this study addresses an important gap in the literature by emphasizing the need for in-depth qualitative exploration of informal payments from multiple viewpoints. This study provides insights that cannot be captured through quantitative methods alone, highlighting its unique contribution to the literature. In doing so, it contributes to both academic literature and practical policy debates on health system governance and reform.

The importance of this research lies not only in identifying the root causes of informal payments, but also in its potential to inform evidence-based interventions and policy reforms. By uncovering the motivations behind these practices and the contextual barriers to eliminating them, this study can assist decision-makers in designing more realistic, culturally sensitive, and sustainable solutions. Furthermore, the findings may be relevant to other health systems facing similar challenges, offering comparative insights for regional and global health policy discussions. Ultimately, addressing informal payments is crucial for building equitable, accountable, and patient-centered healthcare systems, goals that are central to achieving universal health coverage (UHC) and the broader aims of health sector development.

## Methods

### Design

This qualitative study was conducted between September 10, 2024, and January 17, 2025, using a latent inductive content analysis approach [19] to explore both the underlying causes of informal patient payments in hospitals and the strategies for their management from the perspectives of key stakeholders. The research was carried out in multiple hospitals across Iran, representing different sizes, specialties, and ownership types, including both public and private institutions, to capture a broad range of experiences and viewpoints.

### Study area

This study was conducted in Iran, a middle-income country in the Middle East with a mixed public–private healthcare system financed through a combination of government budgets, social health insurance, and out-of-pocket payments. Despite ongoing health sector reforms, including the Health Transformation Plan (HTP), the Iranian health system continues to face challenges related to inequitable financial protection, variability in hospital resources, and differences in service quality between public and private sectors. Hospitals in Iran are categorized into public, private, military, and social security-affiliated institutions, with public hospitals serving the majority of the population, particularly low- and middle-income groups. These hospitals are often characterized by high patient load, resource constraints, and administrative complexity, which may increase vulnerability to informal payment practices. In contrast, private hospitals generally have higher tariffs and more flexible payment structures, potentially influencing patient-provider financial interactions.

Culturally, patient–physician relationships in Iran are influenced by strong social norms of gratitude and gift-giving, which may blur the boundaries between formal and informal payments. In addition, regional disparities in income distribution, insurance coverage, and access to specialized care further contribute to heterogeneity in healthcare experiences across different population groups.

These contextual characteristics make Iran a particularly relevant setting for exploring the drivers and management strategies of informal patient payments in hospitals.

### Participants

A diverse group of stakeholders involved in the experience, governance, or consequences of informal patient payments was purposefully selected using a criterion-based sampling strategy to capture multiple levels of the phenomenon. The selection process began with the identification of key stakeholder categories directly relevant to the research objectives, based on prior literature, expert consultation, and the study’s conceptual framework. Participants included policymakers from the Ministry of Health and Medical Education, selected due to their role in regulation, financing, and national anti-corruption policies; hospital presidents and internal managers, who oversee hospital operations, financial processes, and complaint management systems; medical specialists and nursing managers, as frontline providers who may be directly or indirectly involved in informal payment practices; hospital supervisors affiliated with medical universities, responsible for monitoring hospital performance and compliance; faculty members in health economics and healthcare management, selected for their analytical and policy-oriented perspectives; and patients, who had firsthand experience of making informal payments and could describe the demand-side mechanisms and consequences. Importantly, the inclusion of multiple stakeholder groups was intended to enhance the depth and breadth of understanding of informal payment phenomena across different levels of the health system, allowing for rich data triangulation and a comprehensive exploration of both drivers and management strategies.

### Sampling and sample size determination

In qualitative research, the emphasis is not on sample size or statistical estimation, but rather on the relevance and informational richness of participants in relation to the research objectives [20]. Accordingly, purposive sampling was employed to deliberately recruit individuals who met predefined inclusion criteria and possessed direct knowledge or experience of informal payment practices. Initial participants were identified through professional networks, institutional referrals, and formal correspondence with relevant organizations. Subsequently, a snowball technique was used as a supplementary strategy, whereby interviewed participants suggested other eligible individuals within the predefined stakeholder groups. This approach was used solely to facilitate access to information-rich cases and does not constitute theoretical sampling.

Participant selection was guided by the study objectives rather than by theory generation, and sampling decisions were continuously reviewed to ensure representation across policy, managerial, provider, academic, and patient perspectives. Data collection continued alongside ongoing analysis so that decisions about additional interviews were informed by the emergence of new codes and categories. Data saturation was defined as the point at which no new codes, subcategories, or themes appeared in at least three consecutive interviews, and previously developed categories were fully supported by the data. Saturation was assessed through regular team meetings where the coding progress and thematic development were reviewed. Saturation was reached after interviewing 31 participants, when the last three interviews yielded no new codes and only confirmed existing categories.

### Inclusion and exclusion criteria

Regarding inclusion criteria, participants were included if they had direct and recent involvement in healthcare policy-making, service provision, or receipt of hospital-based healthcare services in Iran, demonstrated awareness of informal payment practices, and were able to engage in in-depth interviews conducted in Persian. For policymakers, managers, and healthcare providers, eligibility required at least two years of continuous experience in their current role within hospital-based or health system governance structures in Iran. For the patient group specifically, inclusion criteria also required having personally made informal payments (cash or in-kind) for hospital-based services such as inpatient care, surgical procedures, or diagnostic tests within the past two months, in either public or private hospitals participating in the study. Regarding exclusion criteria, individuals were excluded if they had no relevant experience within the past two years, declined to give informed consent, refused audio recording of the interview, or were unable to communicate effectively due to cognitive or physical limitations. Patients were additionally excluded if their informal payment experiences were limited to outpatient clinics, to ensure the focus remained on hospital-based contexts.

### Participant characteristics

Participants were diverse in terms of demographic and professional characteristics. The sample included both male and female participants across different age groups (approximately ranging from early 30s to late 60s) and represented various geographical regions across Iran, including major urban and provincial areas. This diversity was considered to ensure a wide range of perspectives across different healthcare contexts.

### Data collection

Data collection was conducted through in-depth, semi-structured interviews using both face-to-face and telephone modalities, depending on the participant group. The data collection instrument (semi-structured interview guide) was developed by the research team as the main tool for data generation. Prior to the main data collection phase, the semi-structured interview guide was carefully developed by the research team based on an extensive review of the relevant literature and aligned with the study objectives, aiming to ensure clarity, relevance, and comprehensiveness of the questions. The interview guide was structured around three main domains: (a) participants’ experiences with informal patient payments in hospital settings, (b) perceived drivers and underlying causes of these payments at individual, organizational, and system levels, and (c) potential strategies and policy recommendations for preventing and managing informal payment practices.

The questions were designed to explore both the drivers of informal patient payments and potential management strategies, using open-ended phrasing to allow participants to share detailed experiences and perspectives. The interview guide was pilot-tested with three individuals from the target population, a physician, a hospital-based service provider, and a health system policymaker, to assess clarity, sequencing, relevance, and appropriateness of probing prompts. Feedback from the pilot led to minor revisions, including rewording certain questions for better comprehension and adding prompts to explore specific contextual issues in more depth. Data from the pilot interviews were excluded from the final analysis but contributed to refining the guide (Table 1).

**Table 1:** Semi-structured interview questions on drivers and management of informal patient payment.

Section	Topic	Main Questions	Probing Question
**1**	**Introduction and Rapport**	Thank participants, explain study purpose, confidentiality, and obtain consent	N/A
**2A**	**Drivers of Informal Payments**	What are the main reasons patients or families make informal payments?	Can you give an example? Why do you think this happens? Which groups are most involved?
		How do informal payments affect patients, providers, and the system?	Consequences for care quality/access? Ethical or cultural considerations?
		What structural, organizational, or systemic factors contribute?	Role of salary/wages, regulations/enforcement?
		What cultural or ethical factors influence informal payments?	Role of gift-giving/social norms? Relation to professional ethics?
**2B**	**Strategies for Managing**	What measures could reduce/eliminate informal payments?	Structural solutions (tariffs, insurance)? Legal/regulatory strategies? Cultural/ethical improvements?
		What challenges/barriers exist in implementing these measures?	Institutional, financial, or social obstacles? How to overcome?
**3**	**Closing**	Any additional experiences or insights?	N/A

Following refinement of the interview guide, the main data collection was carried out by one of the researchers (ARY), who was trained in qualitative interviewing. The interviews were scheduled at times and locations convenient for participants, typically in private offices, meeting rooms within hospitals, or other quiet settings that ensured privacy and comfort. For patients who had been discharged from hospitals, interviews were conducted by telephone to ensure convenience, comfort, and safety. At the beginning of each interview, the study purpose was explained, the importance of the participant’s perspective was emphasized to build trust, and written informed consent was obtained from in-person participants, while verbal consent was obtained from discharged patients during telephone interviews to ensure their agreement and understanding of the study procedures. Participants were assured that their responses would remain confidential and that they could withdraw at any time without consequences.

The interviews were organized into three main sections: an introduction and rapport-building, collection of demographic information such as role, gender, and education level, and the main discussion focusing on the drivers of informal payments and strategies for managing them.

Probing questions such as “Can you give an example?”, “Why do you think this happens?”, and “What would you suggest to solve it?” were used to elicit deeper and more comprehensive insights. Interviews lasted an average of 45 minutes, ranging from 35 to 55 minutes. All interviews were audio-recorded with participant permission, transcribed verbatim in Persian, and securely stored on password-protected devices. Numeric codes were assigned to maintain confidentiality, and any identifying information was removed from transcripts.

### Data analysis

Latent content analysis was used to analyze the data [21]. An inductive approach was adopted, allowing themes and categories to emerge directly from participants’ narratives rather than being pre-specified or theoretically imposed, which is particularly appropriate for exploring sensitive and under-researched phenomena such as informal payments [22]. All audio-recorded interviews were transcribed verbatim in Persian from the original audio files by trained research assistants immediately after each interview. Each transcript was then reviewed and cross-checked against the audio recordings by the primary researcher (ARY) to ensure accuracy and completeness. Data analysis was conducted through an iterative process. Each interview was listened to multiple times, and the corresponding transcript was read line by line to identify meaningful units and key expressions reflecting participants’ perspectives. These units were assigned descriptive open codes, which were continuously compared for similarities and differences. Conceptually similar codes were then grouped into subcategories, which were further merged into broader categories to form the main themes. Throughout the analysis, the coding framework was regularly discussed and refined through research team meetings, and any discrepancies in coding were resolved through consensus. After completing the interviews, the data were analyzed using MAXQDA (version 2022) software.

### Trustworthiness

The rigor of the study was ensured by applying the four established criteria for trustworthiness in qualitative research: credibility, dependability, confirmability, and transferability. Credibility was achieved through prolonged engagement with participants, triangulation of perspectives from different groups, and member checking, whereby summaries of findings were returned to several participants for their review and confirmation. Dependability was ensured by documenting each step of the research process in detail, allowing for replication by other researchers. Confirmability was strengthened by maintaining reflexive notes to minimize researcher bias and by conducting peer debriefing sessions with other members of the research team to review coding decisions and thematic interpretations. Transferability was supported by providing thick descriptions of the study setting, participant characteristics, and data collection procedures, enabling readers to assess the applicability of the findings to other contexts. Confidentiality was maintained by assigning numeric codes to participants, removing identifying information from transcripts, and storing all data securely on password-protected devices. Participants were informed of their right to withdraw from the study at any stage without consequences.

### Ethical considerations

This study is approved by Tehran University of Medical Sciences Ethics Committee with the code of IR.TUMS.SPH.REC.1403.100. All the methods were carried out following relevant guidelines and regulations. Meanwhile, written informed consent (or verbal consent for telephone interviews) was obtained from all the study participants.

## Results

A total of 31 individuals participated in this study, including 3 senior officials from the Ministry of Health and Medical Education of Iran (policymakers), 7 hospital presidents and internal managers, 4 medical specialists, 5 nursing managers, and 2 hospital supervisors from medical universities across Iran. In addition, 5 faculty members specializing in health economics and healthcare management, along with 5 patients who had made informal payments in Iranian hospitals, were also included. Table 2 presents the demographic characteristics of the study participants.

**Table 2:** Characteristics of the study participants.

Row	Variables	Category	Frequency
**1**	**Role**	Policymaker (Senior Official at the Ministry of Health and Medical Education)	3
		Hospital President/Internal Manager	7
		Physician	4
		Nursing Manager	5
		Hospital Supervisor	2
		Faculty Member	5
		Informal Payer	5
**2**	**Gender**	Male	22
		Female	9
**3**	**Education Level**	High School Diploma*	4
		Bachelor’s Degree	7
		Master’s Degree	6
		Ph.D. (Doctor of Philosophy)	8
		Professional Doctorate (e.g., MD)	6

* The high school diploma qualification pertained to the service recipients.

**Fig 1:**
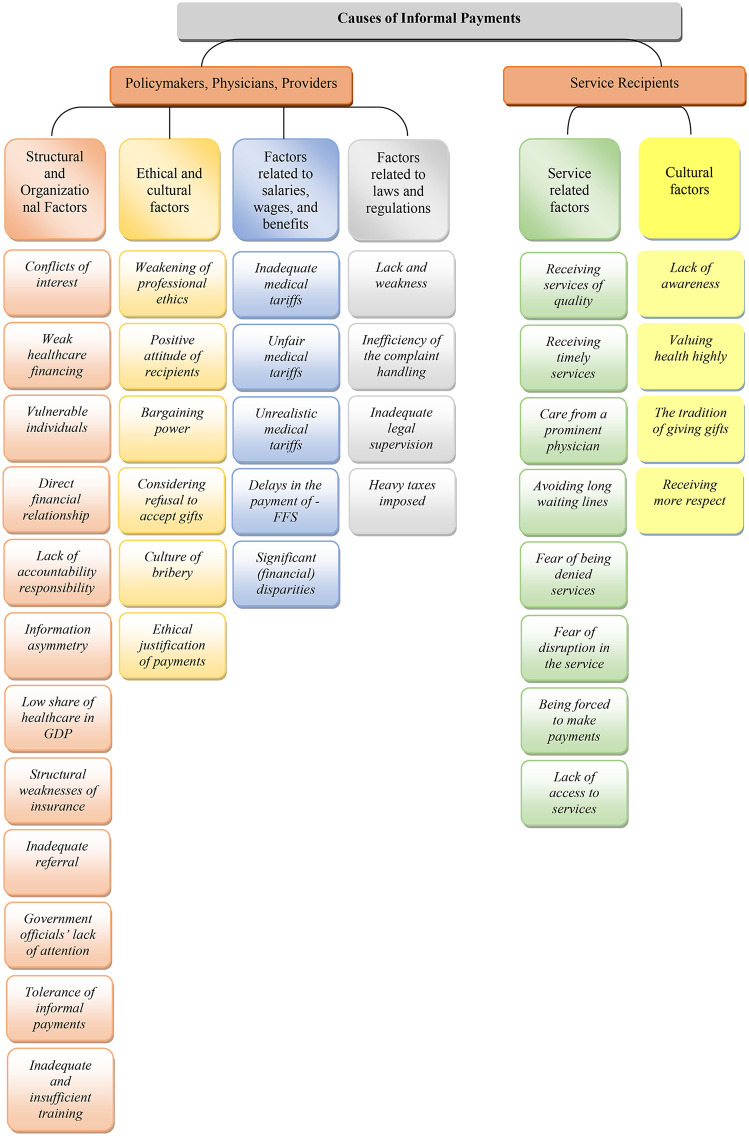
Thematic mapping of informal payment drivers from the perspectives of participants.

In total, six main themes and 39 subcategories were identified regarding the causes of informal patient payments. These six themes include: (a) factors related to salaries, wages, and benefits, (b) structural and organizational factors, (c) legal and regulatory factors, (d) ethical and cultural factors, (e) service-related factors, and (f) patient cultural factors. From the perspective of policymakers, physicians, and healthcare providers, four themes (27 subcategories) were identified, while from the service recipients’ perspective, two themes (12 subcategories) emerged (Table 3, Fig 1).

**Table 3:** Causes of informal payments from the perspectives of policymakers, physicians, providers, and service recipients.

Participants	Main Categories	Sub-categories	Absolute count (n)	Sample quotes
**Policymakers, physicians, and providers**	Factors related to salaries, wages, and benefits	Inadequate medical tariffs	10	*“When tariffs are so low that they don’t even cover our basic expenses, it’s only natural that some turn to informal payments to compensate.”*
		Unfair medical tariffs	8	*“Tariffs are unfairly set for certain specialties; this kind of injustice leads to dissatisfaction and lack of motivation.”*
		Unrealistic medical tariffs	6	*“The tariffs written on paper don’t match the realities of our work. No doctor can provide quality care at these rates.”*
		Delays in the payment of Fee-For-Service (FFS)	7	*“When performance-based payments are delayed for months, many are forced to seek informal income sources just to make ends meet.”*
		Significant (financial) disparities within the medical community	5	*“The income gap between physicians is huge, some earn astronomical sums while others barely get by. This inequality itself fuels informal practices.”*
	Structural and Organizational Factors	Existence of conflicts of interest at the macro level	4	*“Conflicts of interest at high levels of decision-making lead to informal payments being overlooked, or even tacitly encouraged.”*
		Weak healthcare financing	6	*“Insufficient financial resources increase pressure on healthcare providers and create conditions that facilitate informal payment requests.”*
		Lack of support for vulnerable individuals and patients with chronic illnesses	5	*“Vulnerable individuals and patients with chronic conditions are often defenseless against informal payment demands due to a lack of adequate support.”*
		Direct financial relationship between physician and patient	7	*“When there is a direct financial relationship between doctor and patient, the likelihood of informal payments increases.”*
		Lack of accountability and responsibility within the health system	6	*“The lack of clear accountability in the health system contributes to the persistence and expansion of informal payments.”*
		Information asymmetry between service recipients and providers	5	*“When patients lack adequate information about their treatment and associated costs, the risk of exploitation and informal payments rises.”*
		Low share of healthcare in the Gross Domestic Product (GDP)	4	*“The low share of health in the national budget results in resource shortages and financial strain on healthcare workers.”*
		Structural weaknesses of insurance organizations (including deductions from bills and delayed or insufficient reimbursements)	5	*“Insurance system deficiencies, such as delayed reimbursements, impose additional financial burdens and incentivize informal payment practices.”*
		Inadequate referral and tiered healthcare system	4	*“An ineffective referral system leads to overcrowding at higher levels of care, increasing the demand for informal payments.”*
		Government officials’ lack of attention to the status and dignity of healthcare providers	3	*“When the dignity and professional status of healthcare workers are not recognized, their motivation to uphold ethical standards diminishes.”*
		Tolerance of informal payments in the public sector to retain physicians in the system	3	*“The public sector sometimes turns a blind eye to informal payments in order to retain physicians, thereby exacerbating the issue.”*
		Inadequate and insufficient training for healthcare staff	4	*“The lack of adequate training on the consequences of informal payments leaves healthcare staff unprepared to resist or address the phenomenon.”*
	Factors related to laws and regulations	Lack and weakness of restrictive laws and enforcement tools (appropriate punitive system)	5	*“When there are no clear and deterrent penalties for informal payments, there is virtually no serious barrier to the recurrence of this behavior.”*
		Inefficiency of the complaint handling system	4	*“The complaint system is so slow and ineffective that patients prefer not to file a complaint, even if they were forced to make under-the-table payments.”*
		Inadequate legal supervision	3	*“Supervision is mostly superficial; as long as monitoring is not genuine, continuous, and effective, violations will persist.”*
		Heavy taxes imposed on healthcare personnel	3	*“When taxes increase but the actual income of doctors and nurses is not considered, some may turn to informal payments as a way to cope with financial pressure.”*
	Ethical and cultural factors	Weakening of professional ethics	6	*“When ethical commitment in the health system is weakened, boundaries gradually disappear and informal payments become normalized.”*
		Positive attitude of recipients toward informal payments and considering them a rightful compensation (to make up for low salaries)	5	*“Some healthcare workers consider under-the-table payments their right, believing that their salary does not match their workload.”*
		Bargaining power of medical staff over patients	4	*“When patients are compelled to receive care and physicians control the timing and type of treatment, the opportunity for demanding informal payments arises.”*
		Considering refusal to accept gifts (mainly non-cash) by healthcare providers as impolite	3	*“Sometimes, a doctor’s refusal to accept a gift is perceived as disrespectful, which perpetuates gift-giving, even when the intention is not entirely sincere.”*
		A culture of bribery and under-the-table payments in society	4	*“When informal payments are common in other sectors of society, people do not find them unusual in the healthcare system either.”*
		Ethical justification of informal payments by the recipients	3	*“Some healthcare workers try to create ethical justifications for receiving informal payments by citing reasons such as helping the patient or dealing with financial hardship.”*
**Service Recipients**	Service related factors	Receiving services of appropriate quality	5	*“When you pay more, you feel assured that the doctor will spend more time with you and provide better care.”*
		Receiving timely services and saving time	4	*“By making an under-the-table payment, you get an appointment sooner and don’t have to wait for months.”*
		Receiving care from a more prominent physician	3	*“Well-known doctors don’t give appointments without extra money; if you don’t want to pay, you have to see an average doctor.”*
		Avoiding long waiting lines	4	*“Hospital queues are very long; if you pay, your work gets done faster.”*
		Fear of being denied services	3	*“If you don’t pay, you worry that the doctor won’t take proper care of you, or might even turn you away entirely.”*
		Fear of disruption in the service delivery process	3	*“If you don’t offer something, they might suddenly ignore you during treatment or delay your process.”*
		Being forced to make informal payments in order to receive services	4	*“Sometimes, even if you don’t want to pay, the doctor or receptionist acts in a way that makes you feel compelled to give something to get your service.”*
		Lack of access to certain services	3	*“Certain special services are only provided if you pay extra; without that payment, you simply can’t access them.”*
	Cultural factors	Lack of awareness about the illegality of informal payments	3	*“Honestly, I didn’t even know these payments were illegal, I thought it was just the usual way things work.”*
		Valuing health highly (prioritizing health over other concerns)	4	*“When it comes to health, money doesn’t matter; I’m willing to pay whatever it takes to solve the problem.”*
		The tradition of giving gifts as a gesture of gratitude toward healthcare staff	2	*“We’re used to giving something to doctors or nurses as a thank-you, not out of obligation, but out of courtesy and kindness.”*
		Receiving more respect and attention from healthcare providers		*“When you give something, their behavior really changes, they treat you with much more respect.”*

### a. Factors related to salaries, wages, and benefits

Regarding the causes of informal payments, the first main theme was factors related to salaries, wages, and benefits, which included subcategories reflecting financial insufficiencies that drive healthcare providers to seek informal payments (n_=_ 5 subcategories). Participants noted that inadequate, unfair, and unrealistic medical tariffs created substantial financial pressure, leading to informal payment practices. As one physician explained, *“When tariffs are so low that they don’t even cover our basic expenses, it’s only natural that some turn to informal payments to compensate (D1).”* Delays in Fee-For-Service payments and significant income disparities within the medical community were also highlighted as important drivers. A senior official commented, *“The income gap between physicians is huge, some earn astronomical sums while others barely get by. This inequality itself fuels informal practices (P1).”*

### b. Structural and Organizational Factors

The second theme, structural and organizational factors, included weaknesses in the healthcare system and organizational environment (n_=_ 12 subcategories). Subcategories in this theme encompassed conflicts of interest at macro levels, weak healthcare financing, lack of support for vulnerable patients, direct financial relationships between physicians and patients, inadequate accountability, information asymmetry, insufficient insurance structures, and limitations in the referral system. One hospital manager stated, *“When patients lack adequate information about their treatment and associated costs, the risk of exploitation and informal payments rises (H1).”* Participants also emphasized that insufficient training and lack of recognition for healthcare providers’ professional status contributed to the persistence of informal payments.

### c. Factors related to laws and regulations

The third theme, factors related to laws and regulations, reflected the legal environment that either discourages or permits informal payments (n_=_ 4 subcategories). Participants pointed out weak restrictive laws, inefficient complaint handling systems, inadequate legal supervision, and heavy taxation on healthcare personnel. As one policymaker noted, *“When there are no clear and deterrent penalties for informal payments, there is virtually no serious barrier to the recurrence of this behavior (P2).”*

### d. Ethical and cultural factors

The fourth theme, ethical and cultural factors, highlighted societal norms and professional attitudes that normalize informal payments (n_=_ 6 subcategories). Subcategories included the weakening of professional ethics, positive attitudes toward informal payments, bargaining power of medical staff over patients, perception of gift refusal as impolite, societal acceptance of bribery, and ethical justification of informal payments by providers. A nurse remarked, *“When ethical commitment in the health system is weakened, boundaries gradually disappear and informal payments become normalized (N1).”*

### e. Service related factors (Service Recipients’ Perspective)

From the service recipients’ perspective, the first theme was service-related factors, which described motivations for making informal payments to receive timely, high-quality, or preferential care (n_=_ 8 subcategories). Patients reported that informal payments allowed them to access more prominent physicians, avoid long waiting times, and ensure continuity of care. One patient explained, *“Hospital queues are very long; if you pay, your work gets done faster (R1).”*

### f. Cultural factors (Service Recipients’ Perspective)

The second theme, cultural factors, reflected patients’ beliefs, including limited awareness of the illegality of informal payments, prioritization of health over financial considerations, and the tradition of gift-giving as a gesture of gratitude (n_=_ 4 subcategories). As one patient stated, *“Honestly, I didn’t even know these payments were illegal, I thought it was just the usual way things work (R2).”*

Furthermore, regarding practical strategies for managing informal payments, three main themes (structural, legal, and cultural strategies) with 14 subcategories were identified from the participants’ perspectives (Table 4).

**Table 4:** Effective strategies for managing informal payments.

Row	Main Categories	Sub-categories	Absolute count (n)	Sample quotes
**1**	**Structural Strategies**	Making treatment per capita and medical tariffs realistic	7	*“As long as tariffs are not realistic, informal payments will remain a compensatory path for physicians.”*
		Monitoring and controlling standards	6	*“With precise and standard-based supervision, out-of-framework behaviors can be easily identified and addressed.”*
		Eliminating direct financial relationships between physicians and patients	6	*“When there is direct financial exchange between physician and patient, the risk of corruption increases; this relationship must be severed.”*
		Timely payment of claims by insurance organizations	5	*“If insurance organizations pay claims on time, the financial pressure on physicians will decrease, reducing the tendency toward informal payments.”*
		Standardizing professional and ethical behavior	6	*“Training and standardizing professional behavior can strengthen the internalization of ethical values in the health system.”*
		Eliminating conflicts of interest in supervisory systems	4	*“Effective oversight cannot be expected when the supervisor and the stakeholder are the same; conflicts of interest must be seriously addressed.”*
		Strengthening oversight through the creation of clear structures	5	*“Effective monitoring requires clear and institutionalized structures, not ad hoc or discretionary actions.”*
**2**	**Legal Strategies**	Enacting strict and deterrent laws to deal with violators	6	*“If laws are clear and penalties are truly deterrent, many will not dare engage in informal payments.”*
		Self-regulation	5	*“When professional associations take a more active role in regulating their members, the likelihood of violations decreases, as oversight comes from within the medical community itself.”*
		Naming and shaming	4	*If violators are publicly identified and exposed by the media, the resulting shame and social pressure may be even more effective than legal consequences.*
**3**	**Cultural Strategies**	Raising public awareness about patients’ rights	6	*“When patients are aware that they are entitled to proper services without extra payments, they are less likely to accept informal payments.”*
		Changing prevailing beliefs, attitudes, and perceptions in society	5	*“As long as people believe that under-the-table payments are necessary to receive better services, no law alone can eliminate this culture.”*
		Supporting whistleblowers of informal payments	4	*“If someone dares to report a violation but receives no support, not only will their motivation be lost, but others will also lose trust.”*
		Clarifying employees’ legal rights and responsibilities	5	*“When health system employees clearly understand their legal duties and what they are accountable for, the likelihood of misconduct decreases.”*

### a. Structural Strategies

Regarding strategies for managing informal payments, three main themes were identified. Structural strategies (n_=_ 7 subcategories) focused on system-level interventions, such as making medical tariffs realistic, monitoring standards, eliminating direct financial relationships between physicians and patients, timely insurance claim payments, standardizing professional behavior, eliminating conflicts of interest, and strengthening oversight structures. One physician noted, *“As long as tariffs are not realistic, informal payments will remain a compensatory path for physicians (D2).”*

### b. Legal Strategies

Legal strategies (n_=_ 3 subcategories) included enacting strict and deterrent laws, promoting self-regulation through professional associations, and naming and shaming violators. A policymaker emphasized, *“If laws are clear and penalties are truly deterrent, many will not dare engage in informal payments (P3).”*

### c. Cultural Strategies

Cultural strategies (n_=_ 4 subcategories) aimed to change societal attitudes, raise public awareness, support whistleblowers, and clarify employees’ legal rights and responsibilities. A hospital supervisor remarked, *“When patients are aware that they are entitled to proper services without extra payments, they are less likely to accept informal payments (H2).”*

## Discussion

The findings of this study revealed six main themes from the participants’ perspectives regarding the causes of informal payments, including factors related to salaries and benefits, structural and organizational factors, legal and regulatory issues, ethical and cultural factors, service-related factors, and cultural aspects.

Regarding salaries and benefits, low income for healthcare providers, especially physicians, was reported as one of the most important reasons for informal payments. It appears that when payments are low, physicians may exploit their position. In some studies, physicians have stated that the government-determined tariffs are not acceptable, whether in the public or private sector; hence, they demand payments beyond the set tariffs [23]. According to part of the findings by Parsa et al. (2017), unrealistic tariffs were one of the main reasons for under-the-table payments by specialist physicians [24]. In a study by Vian et al. (2006), low salaries of healthcare staff were one of the contributing factors to the prevalence of informal payments in Albania [25]. Khodamoradi et al. (2015) also considered low income among healthcare staff as a reason for such payments [26]. Aryankhesal and Jahangiri (2017) mentioned inadequate oversight of how tariffs are implemented and low wages as major causes of informal payments in Iran [23]. Experts generally view low salaries of healthcare personnel as the main reason for under-the-table payments. Accordingly, some countries implemented official wage increase programs, which had a positive impact on controlling such payments [27]. Evidence from Cambodia, Colombia, the Czech Republic, and Kyrgyzstan shows that changes in payment systems that include salary increases can help reduce informal payments [28]. In Kyrgyzstan, an official salary enhancement plan in two regions significantly reduced informal payments. It appears that low wages inherently create temptations for corruption. When income is insufficient, individuals are forced to take on multiple jobs and may also solicit money from patients. While some countries suggest that higher pay alone is not always necessary to prevent corruption, paying a wage appropriate to working conditions can indeed deter informal payments [27]. It appears that low wages and unrealistic tariffs are not merely an economic problem; rather, they contribute to the formation of a moral justification among some healthcare providers. When physicians and healthcare staff perceive that official remuneration is not commensurate with their responsibilities and workload pressures, the acceptance of informal payments may be interpreted as a means of correcting structural injustice. This interpretation is supported by recent evidence from Iran, indicating that informal payments are often rooted in systemic dissatisfaction with financial policies rather than merely in individual tendencies toward corruption [29].

Another cause identified was structural and organizational factors. Amiresmaili et al. (2013) found structural issues such as the direct physician-patient relationship, a low share of health in GDP, discrimination within the medical community, delays in insurance reimbursements, inappropriate insurance policies, weak referral and service-leveling systems, and inefficiencies in complaint mechanisms as major contributors to informal payments by physicians [30]. According to Parsa et al. (2017), incomplete insurance coverage was one of the main drivers of under-the-table payments [24]. Khodamoradi et al. (2015) cited factors like surplus of physicians, hospital beds and medical staff, poor public awareness of basic health services, weak performance by public purchasers and insurance organizations as structural contributors [26]. Aryankhesal and Jahangiri (2017) also pointed to weaknesses in the health system, including ineffective insurance financing, unreasonable deductions by insurance organizations, and asymmetric information between providers and recipients as key factors [23]. Informal payments disrupt access to healthcare, reduce system efficiency, and serve as a barrier to health reforms [31]. The interpretation of the findings indicates that structural weaknesses, such as inefficiencies in the referral system, incomplete insurance coverage, and delays in reimbursement, shift institutional pressures to the level of direct interaction between patients and service providers. Under such conditions, informal payments become an unofficial mechanism for managing resource shortages and systemic uncertainty. This suggests that informal payments are less the result of individual choice and more a consequence of organizational and managerial failures within the health system [32].

Regarding legal and regulatory factors, several studies identified the absence of restrictive laws, effective penalties, and enforcement mechanisms as contributing to informal payments in health systems [33–39]. Amiresmaili et al. (2013) highlighted inadequate legal oversight and weak legislation as key factors [30]. Khodamoradi et al. (2015) pointed to weak oversight in the health system, lack of regulations, and poor government accountability as drivers [26]. Other research also emphasized poor legislation, weak enforcement, lack of supervision, and absence of strict consequences for those receiving informal payments as main reasons [40]. In their study, Amiresmaili et al. (2013) encountered examples where patients, despite knowing that extra payments were unjustified, still made them because they believed that both the law and complaint mechanisms were ineffective. Patients noted that they could not file complaints due to the lack of evidence, and some feared receiving lower-quality care if they complained [30]. The findings suggest that the absence of effective deterrent regulations and weaknesses in oversight mechanisms lead to a low perceived risk of misconduct among recipients of informal payments. Even when patients are aware of the illegality of such payments, the lack of institutional support and fear of negative consequences associated with filing complaints discourage them from using formal reporting mechanisms. This situation creates a profound gap between written law and patients’ lived experiences, thereby perpetuating the practice of informal payments.

Ethical and cultural factors were also found to be influential. Khodamoradi et al. (2015) [26], Tomini et al. (2010) [41], Farcasanu et al. (2010) [36], Belli et al. (2004) [23], Shahriari et al. (2001) [42], and Amiresmaili et al. (2013) noted that cultural norms can drive informal payments. Some service recipients believe that when someone does a good deed for you, you should reciprocate with generosity [30]. In several studies, gratitude-related payments to physicians were commonly given as gifts after treatment [31,36,43,44]. Additionally, health is perceived in Iranian culture as a critical personal asset, and people are willing to pay to preserve or restore it. Another cultural factor is Iran’s widespread “modesty culture,” which makes people reluctant to say “no” to requests, even when they know they are unlawful, especially when requests come from physicians. The interpretation of the findings indicates that cultural norms, such as reciprocating favors and the high value placed on health in Iranian culture, can transform the receipt of informal payments from an overt misconduct into a behavior perceived as understandable by some actors. However, evidence suggests that targeted education in bioethical principles can reshape healthcare workers’ ethical attitudes and reduce the acceptance of unethical behaviors, including the receipt of informal payments. This highlights that ethics-based interventions are a necessary complement to structural reforms [45].

Service-related factors were another driver of informal payments. Amiresmaili et al. (2013) reported that patients often made informal payments to access better or more famous physicians, receive higher-quality care, avoid treatment delays, skip queues, or ensure their care wasn’t assigned to students. They also found that some physicians who demanded additional payments actively cultivated the perception among patients that they were superior and offered higher-quality care [30]. According to Vian et al. (2006), the desire to receive better services was a key factor behind informal payments in Albania [25]. A study by Özgen et al. (2010) in Turkey showed that 31% of patients made informal payments to healthcare workers [31]. Aryankhesal and Jahangiri (2017) cited the pursuit of faster, more respectful, and higher-quality services as reasons for informal payments in Iran [23]. Some patients reported making such payments out of fear of receiving poor or substandard services. One study found that 42% of patients paid informally due to concerns about inadequate care [44]. Many patients made informal payments to avoid long waiting times or get off surgical waiting lists. A study in three Baltic countries (Latvia, Lithuania, and Estonia) showed that more than half of respondents were willing to pay hospitals in exchange for faster treatment [44–47]. From the authors’ perspective, informal payments in this theme reflect patients’ risk-management behaviors. Faced with long waiting lines, resource shortages, and uncertainty regarding the quality of services, patients perceive informal payments as a means to secure the desired level of care. This pattern is particularly pronounced in crisis situations, such as the COVID-19 pandemic, where workload pressures and weaknesses in healthcare service management can exacerbate inequalities in access [48].

Another section of the study focused on strategies for managing and controlling informal payments, resulting in three main themes: structural, legal, and cultural approaches.

From a structural perspective, one strategy is to guarantee provider compensation through government funding. Informal payments often reflect resource shortages in the health sector. One way to reduce this gap is to increase allocated health funding. Some transitioning countries have developed insurance plans funded by payroll taxes to boost health budgets [26]. Another strategy is to reform payment mechanisms. Replacing the outdated Fee-For-Service (FFS) model, which creates direct financial ties between patients and providers, with alternatives like capitation (per capita payments), which sever such ties, can be effective. Making tariffs realistic while strengthening insurance institutions is critical. When tariffs are below actual service costs, providers and even health authorities may view under-the-table payments as a necessary workaround [23].

On the one hand, the strategy of managing informal payments through legal instruments can be implemented by regulating laws to prevent abuse of power. Such regulation can be achieved through strategies like monitoring and standard control, self-regulation (by professional groups), incentives and market-based controls (public motivation for good behavior), naming and shaming offenders, and establishing legal rights and accountability, all of which are among the policies to combat informal payments [26]. Moreover, according to another study, strict and well-planned laws against informal payments in the form of fines, reprimands, and dismissals can effectively reduce their prevalence [40]. On the other hand, formalization may help distinguish between payments that are essentially life-saving responses and those driven by abuse of power. However, a punitive approach is only effective when staff believe the punishments are severe enough, so the cost of accepting informal payments outweighs the benefit. If salaries are low and the main penalty for accepting under-the-table payments is job loss, this could lead to staff leaving the sector altogether. To address this issue, official salaries must be increased [26]. It seems that the effectiveness of legal approaches in controlling informal payments depends on the simultaneous implementation of deterrent regulations and the provision of sustainable remuneration for staff. Without financial reforms and job security measures, purely punitive policies may lead to workforce attrition or the concealment of misconduct. Therefore, informal payments, rather than being merely legal violations, reflect the unequal interplay of power, oversight, and job security within the health system.

Cultural factors can also play a strategic role in managing informal payments. One of the policies to counteract informal payments is to change prevailing perceptions and beliefs in society. In this regard, in Albania, a major part of public education efforts to combat informal payments has focused on people’s ethical beliefs [25]. However, findings from two studies conducted in Iran indicate that people already believe that informal payments are wrong [15,49]. Therefore, efforts to convince people that informal payments are a form of corruption may not significantly reduce the practice.

Instead, behavior-change strategies should focus on breaking the chain of belief that says “you must make informal payments to receive high-quality care, and if you don’t, you will be mistreated.” This is only achievable when people are convinced that medical staff are adequately compensated through official channels, so they can receive quality care without making informal payments, and also that healthcare staff are penalized for soliciting extra payments from patients [25]. Qualitative data from a study in Albania show that people may feel obliged to make financial payments because they believe the staff receive insufficient official compensation [50]. Another strategy is to publicly announce quality improvement programs, which can help convince the public that they are receiving appropriate high-quality services without the need for informal payments. Finally, considering penalties for physicians and others who insist on receiving informal payments can help create the perception that if a doctor is punished for demanding unofficial fees, others will refrain from such practices as well [25]. The findings indicate that public awareness of the illegitimacy of informal payments alone is insufficient to change behavior. These results may suggest that deeply rooted beliefs in the necessity of payments to receive quality care play a central role in perpetuating this phenomenon. As long as public trust in fair staff remuneration and the effective enforcement of regulations is not strengthened, informal payments will continue to serve as an adaptive strategy in patients’ behavior.

### Limitations and strengths

This study has several limitations that should be acknowledged. First, the sensitive and often concealed nature of informal payments posed challenges for data collection, particularly in obtaining accurate and candid accounts from those directly involved in receiving such payments. As a result, certain key perspectives, especially those of healthcare providers who engage in informal transactions, may be underrepresented. Second, the lack of reliable national data or systematic reporting on the prevalence and magnitude of informal payments limited our ability to contextualize the findings within broader quantitative trends. Finally, while qualitative methods are well-suited for exploring complex social phenomena, the findings may not be generalizable beyond the specific institutional and cultural context in which the study was conducted.

However, the study also has notable strengths. The in-depth qualitative approach provided rich, firsthand insights into participants’ experiences and perceptions, allowing us to uncover nuanced factors influencing informal payments in healthcare. Moreover, the rigorous coding and theme extraction process enhances the credibility and trustworthiness of the findings.

### Suggestions for future research

Future research should aim to explore the prevalence and financial scale of informal patient payments through robust quantitative methods, including nationally representative surveys. Such data could help policymakers better understand the scope of the issue and evaluate the effectiveness of existing regulatory efforts. In addition, longitudinal and mixed-methods studies are recommended to examine how informal payment practices evolve over time and in response to policy interventions. Further research should also focus on the perspectives of healthcare providers who engage in informal transactions, as their motivations and constraints remain understudied due to access limitations. Comparative studies across different cultural and institutional contexts could also provide valuable insights into how socio-political environments influence the drivers and control of informal payments. Finally, intervention-based research, testing specific strategies such as public education campaigns, financial incentive models, or enforcement mechanisms, would contribute to evidence-based policy design aimed at reducing informal payments in healthcare systems.

### Implications and applications

The results of this study have important implications for health system governance, transparency, and accountability. By identifying the root causes of informal payments, health policymakers can better design context-specific interventions aimed at strengthening public trust in the healthcare system. The findings also offer practical guidance for hospital managers and health administrators on developing internal monitoring systems, incentivizing ethical behavior among staff, and engaging the public in awareness campaigns. Additionally, the study may inform training and capacity-building programs for healthcare providers to reinforce professional standards and discourage informal transactions.

## Conclusion

This qualitative study sheds light on the complex and multifaceted drivers of informal patient payments in hospital settings. Among the various factors identified, low staff remuneration, structural and organizational weaknesses, and cultural and ethical norms emerged as the most frequent and influential causes across participants. These dominant themes indicate that informal payments are not solely the result of individual misconduct but are deeply rooted in systemic issues and societal expectations. Addressing these key drivers requires a comprehensive, multi-level policy approach targeting both the supply and demand sides of informal transactions. Implementing such interventions is essential for improving health system equity, enhancing trust, and ensuring overall service quality. By highlighting the three main causes, this study provides actionable insights for policymakers, hospital managers, and health administrators. These insights can help design context-specific interventions, strengthen legal and institutional frameworks, enhance public awareness, and foster a culture of ethical practice among healthcare providers.

### Policy recommendations

Based on the findings of this study, a set of targeted policy actions is proposed to address the root causes of informal patient payments and support the implementation of effective management strategies within the healthcare system:

***Strengthen Legal and Regulatory Frameworks***: Implement and enforce clear laws prohibiting informal payments, with proportionate penalties that deter both providers and patients from engaging in such practices.

***Improve Healthcare Worker Compensation***: Increase formal salaries and performance-based incentives to reduce the financial pressure that may lead providers to seek unofficial income.

***Enhance Transparency and Accountability Mechanisms***: Establish robust monitoring and reporting systems within hospitals, including anonymous complaint channels for patients.

***Promote Public Awareness***: Launch targeted education campaigns to reshape public perceptions and reduce the social acceptability of informal payments.

***Encourage Cultural Change Within Institutions***: Support internal initiatives that foster a culture of integrity and ethical conduct among healthcare staff through training, peer support, and leadership engagement.

***Evaluate and Scale Successful Interventions***: Pilot and rigorously assess anti-corruption programs or payment reforms, then scale effective models nationally.
